# Case Report: Strangulated intestinal obstruction due to chronic migration of an intrauterine device (IUD): a 30-year latent complication

**DOI:** 10.3389/fmed.2025.1613116

**Published:** 2025-07-07

**Authors:** Hong-Xia Song, Tian-Hao Xie, Yan Fu, Xiao-Shi Jin, Qiang Wang, Zheng Niu, Qian Sun, Xiu-Hua An

**Affiliations:** ^1^Department of General Surgery, Affiliated Hospital of Hebei University, Baoding, Hebei, China; ^2^Basic Research Key Laboratory of General Surgery for Digital Medicine, Affiliated Hospital of Hebei University, Baoding, Hebei, China; ^3^Department of Ophthalmology, Baoding No. 1 Central Hospital, Baoding, Hebei, China; ^4^Department of Internal Medicine, Wangdu County Chinese Medicine Hospital, Baoding, Hebei, China

**Keywords:** intrauterine device (IUD), strangulated intestinal obstruction, migration, uterine perforation, case report

## Abstract

Intrauterine devices (IUDs) are widely used but carry rare risks of migration and subsequent complications, such as bowel obstruction. This case highlights the life-threatening potential of chronic IUD migration decades after insertion, emphasizing the need for heightened clinical vigilance and long-term surveillance. A 57-year-old female patient presented to the hospital with a 2-day history of abdominal pain, accompanied by the cessation of flatus and defecation. She had one pregnancy and one vaginal delivery 31 years ago, followed by the insertion of a ring-shaped IUD 1 year postpartum. However, she had not undergone any follow-up examinations since the IUD placement. Two years prior to admission, she attempted to have the IUD removed, but it was not detected within the uterine cavity. This resulted in the assumption that the device had been spontaneously expelled, and no further investigations were pursued at that time. CT imaging revealed small bowel obstruction and a ring-shaped intra-abdominal foreign body. Emergency laparotomy identified a migrated IUD strangulating 100 cm of necrotic ileum. Upon exploration of the uterus, a fibroid was identified on the posterior wall, but no acute perforations or other pathological changes were noted. Subsequently, the IUD was removed, and bowel resection with anastomosis was performed. Chronic IUD migration may evade detection for decades, culminating in catastrophic bowel obstruction. Clinicians must maintain high suspicion for IUD-related complications in patients with abdominal pain, even years after insertion. Prophylactic removal of misplaced devices and long-term imaging surveillance are critical to prevent morbidity. Early recognition of such rare but severe complications through comprehensive clinical assessment and imaging studies can significantly improve patient outcomes and reduce the risk of life - threatening bowel - related events.

## 1 Introduction

Intrauterine devices (IUDs) are among the most widely used long-acting reversible contraceptives globally, valued for their high efficacy (>99%) and cost-effectiveness (1). Despite their widespread use and safety profile, rare complications such as uterine perforation and subsequent migration into the abdominal cavity pose significant clinical challenges, with an estimated incidence of 0.3–2.6 per 1,000 insertions (2). Among these complications, small bowel obstruction caused by migrated IUDs is exceptionally rare but carries life-threatening risks due to delayed diagnosis and the potential for bowel strangulation (3). Since the year 2000, there have been 14 case reports published in China regarding strangulated intestinal obstruction (SIO) caused by IUDs, while only 5 English-language case reports (intestinal necrosis confirmed by operation) on this topic have been published internationally (4–8) (Table 1).

**TABLE 1:** Overview of documented cases.

References	Patient age	Parity	Type of IUD	Symptoms	Time to diagnosis	Time between placement and diagnosis
Li et al. (4)	59	Gravida 2, para 2	Loop	Abdominal pain	1 day	20 years
Yang and Zhou (5)	77	NM	Loop	Peritonitis signs	2 days	Over 30 years
Xu et al. (6)	79	NM	NM	Epigastric pain	A few hours	Over 50 years
Mellow et al. (7)	63	NM	NM	Abdominal pain and vomiting	A few hours	30 years
Zheng et al. (8)	72	NM	Loop	Upper abdominal pain and vomiting	3 days	NM
Personal case	57	Gravida 1, para 1	Loop	Abdominal pain	2 days	30 years

NM, not mentioned.

Current guidelines on IUDs management, such as those published by the U.S. Selected Practice Recommendations for Contraceptive Use in 2016 and 2024, emphasize the high efficacy and safety of IUDs but do not uniformly advocate for routine follow-up visits for asymptomatic, healthy women after IUD placement (9, 10). These recommendations stem from the scarcity and low quality of evidence concerning the influence of follow-up timing on IUD effectiveness and the occurrence of complications. However, the limited evidence base and the lack of clear guidelines on long-term surveillance for IUD users, particularly those with devices in place for extended periods, leave a gap in clinical practice regarding guiding strategies. Reports of IUD displacement and complications years after insertion suggest that more comprehensive follow-up protocols may be warranted, especially for individuals who have retained an IUD for over a decade (1). Herein, we report a case of SIO caused by a migrated IUD 30 years post-insertion. This case highlights the importance of recognizing IUD-related complications and synthesizes current evidence on the mechanisms, diagnostic strategies, and management approaches. Furthermore, we aim to emphasize the need for heightened clinical vigilance and standardized follow-up protocols to mitigate long-term risks associated with IUD use.

## 2 Case report

A 57-year-old female patient presented to the hospital with a 2-day history of abdominal pain, accompanied by the cessation of flatus and bowel movements. The pain initially localized to the periumbilical region and later became diffuse, involving the entire abdomen. The pain was persistent and unrelieved, associated with the absence of anal flatus and defecation.

The patient had a medical history of hypertension for 6 years, managed with irbesartan tablets, with well-controlled blood pressure. She also reported a history of pleurisy 10 years prior, which had resolved completely, and hyperthyroidism 5 years ago, which was stabilized with oral antithyroid medication and subsequently discontinued. Obstetrically, she had one pregnancy and one vaginal delivery 31 years ago, followed by the insertion of a ring-shaped IUD 1 year postpartum. However, she had not undergone any follow-up examinations since the IUD placement. Two years prior to admission, she attempted to have the IUD removed, but it was not visualized within the uterus. This led to the assumption that the device had been spontaneously expelled, and no further investigations were pursued at that time.

Physical examination revealed abdominal distension without visible gastrointestinal peristaltic waves or abdominal wall varicosities. Bowel sounds were absent, and no vascular murmurs were auscultated. Diffuse tenderness and rebound tenderness were present, with localized muscle rigidity in the periumbilical region. The liver and spleen were non-palpable, and no abdominal masses were detected. Abdominal percussion elicited tympany, and shifting dullness was negative.

**FIGURE 1:**
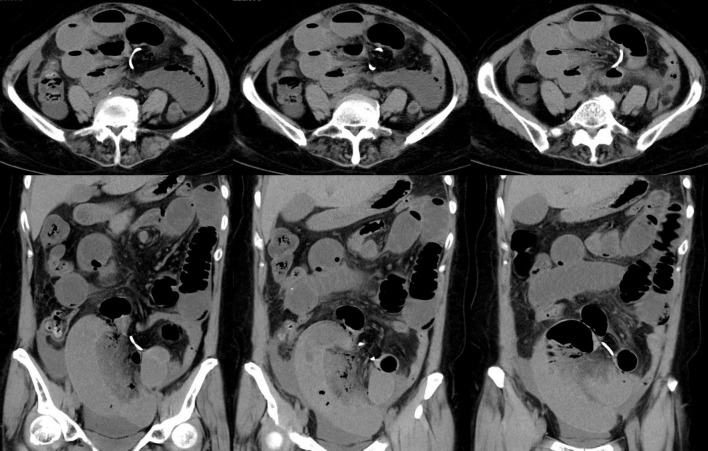
CT imaging demonstrated a ring-shaped high-density shadow in the abdominal cavity.

CT imaging demonstrated gas and fluid accumulation in the small intestine, along with free fluid in the abdominal and pelvic cavities. A ring-shaped high-density shadow was identified in the abdominal cavity (Figure 1). No abnormal densities were observed in the bilateral adnexal regions or the uterus. Laboratory tests revealed the following findings: a white blood cell count of 9.47 × 10^9^/L, hemoglobin level of 105 g/L, neutrophil count of 7.33 × 10^9^/L, C-reactive protein level of 180.5 mg/L, and procalcitonin level of 0.730 ng/mL. Hemorrhagic fluid was aspirated during diagnostic abdominal paracentesis.

**FIGURE 2:**
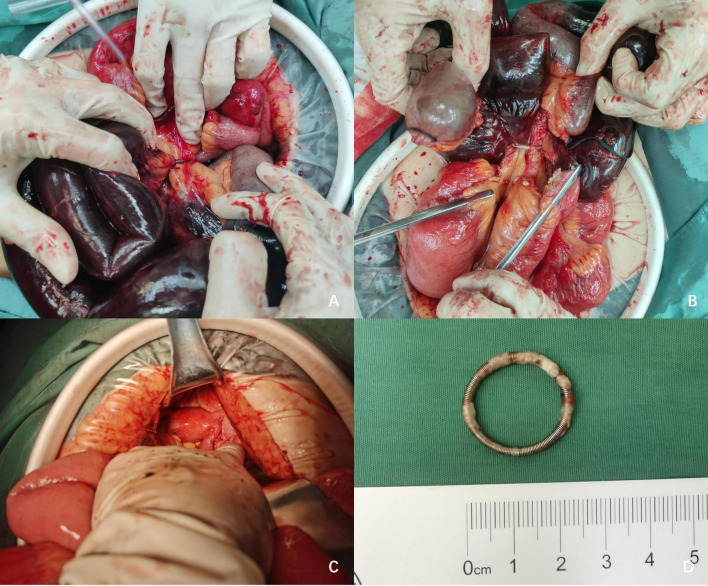
**(A)** The mid-segment of the small intestine and its mesentery were found to be entrapped within a copper ring-shaped IUD. **(B)** The strangulated small intestine was severed. **(C)** No acute perforations or other pathological changes were noted upon exploration of the uterus. **(D)** IUD.

Given the clinical and imaging findings, SIO was suspected, prompting an exploratory laparotomy. Intraoperatively, approximately 300 mL of hemorrhagic fluid was observed in the abdominal cavity. The mid-segment of the small intestine and its mesentery were found to be entrapped within a copper ring-shaped IUD, resulting in strangulation and necrosis of approximately 100 cm of the small intestine (Figures 2A, B). The remainder of the small intestine and colon appeared normal, with no evidence of space-occupying lesions. Upon exploration of the uterus, a fibroid was identified on the posterior wall, but no acute perforations or other pathological changes were noted (Figure 2C). The necrotic segment of the small intestine was resected, and the IUD was removed (Figure 2D). Following intestinal decompression, a side-to-side anastomosis was performed, and drainage tubes were placed in the abdominal and pelvic cavities for postoperative management.

Postoperatively, the patient was administered comprehensive symptomatic treatment, including antibiotics to prevent infection, acid-suppressive therapy to reduce gastric acid secretion, analgesics for pain management, as well as fluid replacement and nutritional support to promote recovery. On the third postoperative day, the patient passed flatus and had her first bowel movement. She was advised to maintain adequate hydration. By the fifth day, she had transitioned to a liquid diet, and the drainage tubes were removed on the seventh day. The patient’s recovery was uneventful, and she was discharged on the ninth postoperative day.

Follow-up evaluations at 2 and 6 months postoperatively demonstrated normal gastrointestinal function, with no abnormalities detected on abdominal CT imaging. Throughout the follow-up period, the patient reported high satisfaction with the treatment outcomes. The timeline is described in Table 2.

**TABLE 2:** The timeline of the diagnosis and treatment process.

Timeline	Diagnosis and treatment process
1994	A ring-shaped IUD was placed
Apr 13th, 2022	IUD was not found in the uterus
Feb 26th, 2024	Abdominal pain accompanied by the cessation of flatus and bowel movements
Feb 28th, 2024 (night time)	Pain intensified, and the patient was hospitalized in the department of General Surgery
Feb 29th, 2024 (early hours)	Surgery was performed, and the condition known as SIO caused by the IUD was confirmed
Mar 3th, 2024	Patient passed flatus and had her first bowel movement
Mar 5th, 2024	Patient started a liquid diet
Mar 7th, 2024	Drainage tubes were removed
Mar 9th, 2024	Patient was discharged from the hospital

IUD, intrauterine device; SIO, strangulated intestinal obstruction.

## 3 Discussion

Intrauterine device migration into the abdominal cavity is a rare but potentially catastrophic complication, with two primary mechanisms: acute perforation during insertion and chronic transmural migration post-placement. Acute perforation typically occurs due to technical errors, uterine anomalies, or inadequate patient selection. Risk factors include uterine anomalies such as retroversion or retroflection, operator inexperience, and postpartum insertion, particularly within 6 weeks of delivery when the uterine wall is thinner and more fragile (2, 11). In the present case, the absence of uterine scarring or acute symptoms at the time of insertion suggests that acute perforation was unlikely. However, the patient’s history of IUD placement shortly after childbirth (1 year postpartum) may have contributed to subclinical myometrial weakening, facilitating later migration.

Chronic migration, on the other hand, is a gradual process driven by uterine contractions and mechanical forces acting on the IUD over time. This mechanism is particularly relevant in cases where the IUD is initially correctly positioned but later migrates into the peritoneal cavity. Repetitive uterine contractions, especially during menstruation or labor, can exert forces of up to 50 Newtons (N), sufficient to erode the myometrium (12). Additionally, the IUD incites a chronic inflammatory response, leading to fibrosis and adhesion formation. Over time, these adhesions may anchor the device to adjacent structures such as the bowel or omentum (1). Device design also plays a critical role; closed-ring IUDs (e.g., Antigon, Chinese stainless steel rings) are more prone to migration due to their rigid structure and lack of flexibility (13). In this case, the ring-shaped IUD migrated into the peritoneal cavity over 30 years, eventually causing ileal herniation and necrosis. The absence of uterine perforation scars indicates that the device gradually eroded through the myometrium, likely facilitated by chronic inflammation and adhesions. Another possibility was that the IUD might have been initially misplaced or might have experienced positional changes post - insertion, migrating from the thinnest part of the cornual region to an extrauterine location. This is an important consideration for clinicians, as it underscores the potential for delayed presentation of complications.

Once the IUD breaches the uterine wall, it may migrate to various intra-abdominal locations, including the omentum, bowel, bladder, and pelvic sidewalls (14). The most common complications include bowel obstruction, visceral perforation, and adhesion formation. Migrated IUDs can entrap bowel loops, leading to mechanical obstruction and ischemia. Closed-ring devices are particularly hazardous due to their ability to form a complete loop around the bowel (15). Chronic inflammation around the IUD can lead to dense adhesions, complicating surgical retrieval (16). In this patient, the IUD migrated to the mid-abdomen, where it entrapped a segment of ileum, causing strangulation and necrosis. The absence of acute symptoms until bowel obstruction highlights the insidious nature of chronic migration.

Despite advancements in IUD design and imaging techniques, challenges persist in early detection. Up to 31% of migrated IUDs are asymptomatic (1). Imaging plays a critical role in diagnosing migrated IUDs and assessing complications. Ultrasound is the first-line modality for routine follow-up but fails to detect 40%–50% of extrauterine devices, especially non-echogenic levonorgestrel IUDs (17). CT is the gold standard for locating migrated IUDs and identifying secondary complications such as bowel obstruction or abscess formation (18). MRI is useful for evaluating soft tissue involvement but is less commonly used due to cost and availability (19). In this case, CT identified the migrated IUD as a hyperdense ring in the mid-abdomen, with associated small bowel dilation and mesenteric fat stranding. This finding enabled timely surgical intervention, preventing further complications.

Prevention strategies focus on device design improvements, insertion technique optimization, and long-term surveillance. Modern frameless or flexible IUDs (e.g., GyneFix^®^) reduce perforation risks, but their adoption remains limited in regions using legacy devices (1, 12, 20). Transition to newer designs with lower migration potential is critical. Biomaterials, particularly nanomaterials, show tremendous potential for future advancements in contraception (21). Ultrasound guidance during insertion reduces perforation rates by 60% in high-risk patients, such as those with retroverted uteri or postpartum (22). To minimize the risk of perforation, it is advisable to avoid placing an IUD during lactation and within the first 36 weeks postpartum (23).

Although clinical practice guidelines do not recommend routine follow-up visits for asymptomatic, healthy women after IUD placement, patients can conduct self-checks to confirm the presence of the IUD strings (9, 10). While the clinical utility of this approach is limited, it can help reduce unnecessary clinic visits (24). Conversely, the appearance of unexpected symptoms or signs suggestive of IUD expulsion may enable patients to self-identify potential issues and seek follow-up care (25). Nevertheless, reports of IUD displacement and additional research suggest that patients may benefit from annual routine examinations aimed at preventing potential complications (1). This is particularly relevant for individuals who have had an IUD in place for over a decade; annual imaging studies, such as ultrasound or X-ray examinations, are recommended for this group (26). Patient education is also critical, emphasizing the importance of reporting abdominal pain or missing threads (27).

## 4 Conclusion

This case report describes a 57-year-old female who developed SIO 30 years after the insertion of a copper IUD, which had migrated into the abdominal cavity. The case highlights several unique aspects: (1) an exceptionally long latency period between IUD insertion and symptomatic presentation, (2) the involvement of a loop-shaped copper device—a rare cause of such complications—and (3) the absence of acute uterine perforation findings, suggesting chronic migration. This case underscores the life-threatening potential of migrated IUDs, emphasizing the need for heightened clinical suspicion in patients presenting with abdominal pain and a history of IUD use. Chronic migration may evade detection for decades, necessitating thorough imaging evaluation. Prophylactic removal of misplaced IUDs, even if asymptomatic, is crucial to prevent catastrophic complications. Future studies should focus on improving device designs and standardizing follow-up protocols to mitigate long-term risks.

## Pubslisher’s note

All claims expressed in this article are solely those of the authors and do not necessarily represent those of their affiliated organizations, or those of the publisher, the editors and the reviewers. Any product that may be evaluated in this article, or claim that may be made by its manufacturer, is not guaranteed or endorsed by the publisher.

## Data Availability

The original contributions presented in this study are included in this article/supplementary material, further inquiries can be directed to the corresponding author.
